# Role of Electrostatic Interactions in Calcitonin Prefibrillar Oligomer-Induced Amyloid Neurotoxicity and Protective Effect of Neuraminidase

**DOI:** 10.3390/ijms22083947

**Published:** 2021-04-11

**Authors:** Ida Cariati, Roberto Bonanni, Mario Marini, Anna Maria Rinaldi, Beatrice Zarrilli, Virginia Tancredi, Claudio Frank, Giovanna D’Arcangelo, Marco Diociaiuti

**Affiliations:** 1 Medical-Surgical Biotechnologies and Translational Medicine (Phd), Department of Clinical Sciences and Translational Medicine, “Tor Vergata” University of Rome, Via Montpellier 1, 00133 Rome, Italy; 2 Department of Systems Medicine, “Tor Vergata” University of Rome, Via Montpellier 1, 00133 Rome, Italy; roberto.bonanni1288@gmail.com (R.B.); mario.marini@uniroma2.it (M.M.); annamaria.rinaldi@uniroma2.it (A.M.R.); b.zarrilli@outlook.it (B.Z.); tancredi@uniroma2.it (V.T.); giovanna.darcangelo@uniroma2.it (G.D.); 3 Centre of Space Bio-Medicine, “Tor Vergata” University of Rome, Via Montpellier 1, 00133 Rome, Italy; 4 UniCamillus-Saint Camillus International University of Health Sciences, Via di Sant’Alessandro 8, 00131 Rome, Italy; claudio.frank@unicamillus.org; 5 Centro Nazionale Malattie Rare, Istituto Superiore di Sanità, 00161 Rome, Italy; marco.diociaiuti@iss.it

**Keywords:** amyloid neurotoxicity, salmon calcitonin, soluble prefibrillar oligomers, lipid rafts, GM1, neuraminidase, cell viability, synaptic transmission, neurodegeneration

## Abstract

Salmon calcitonin is a good model for studying amyloid behavior and neurotoxicity. Its slow aggregation rate allows the purification of low molecular weight prefibrillar oligomers, which are the most toxic species. It has been proposed that these species may cause amyloid pore formation in neuronal membranes through contact with negatively charged sialic acid residues of the ganglioside GM1. In particular, it has been proposed that an electrostatic interaction may be responsible for the initial contact between prefibrillar oligomers and GM1 contained in lipid rafts. Based on this evidence, the aim of our work was to investigate whether the neurotoxic action induced by calcitonin prefibrillar oligomers could be counteracted by treatment with neuraminidase, an enzyme that removes sialic acid residues from gangliosides. Therefore, we studied cell viability in HT22 cell lines and evaluated the effects on synaptic transmission and long-term potentiation by in vitro extracellular recordings in mouse hippocampal slices. Our results showed that treatment with neuraminidase alters the surface charges of lipid rafts, preventing interaction between the calcitonin prefibrillar oligomers and GM1, and suggesting that the enzyme, depending on the concentration used, may have a partial or total protective action in terms of cell survival and modulation of synaptic transmission.

## 1. Introduction

The protein accumulation in the form of amyloid fibrils is a common feature of many neurodegenerative diseases [1,2]. Although proteins involved in these diseases are not related to each other in structure or function, amyloid aggregates have surprisingly similar characteristics, including a high propensity for abnormal folding and a tendency to self-aggregate [3,4,5].

It is generally accepted that cytotoxic species are low molecular weight soluble Prefibrillar Oligomers (PFOs) [6]; however, the mechanisms by which they trigger toxicity and neuronal death processes have yet to be clarified. In fact, due to the rapid rate of aggregation and their structural instability, it is not known which aggregation species are neurotoxic and what is the real mechanism of action. For this reason, it is common to use experimental models that, following aggregation, form PFOs simulating the toxic effect of amyloid proteins [7,8,9,10]. In particular, we use salmon Calcitonin (sCT), which is characterized by a slow rate of aggregation, a property that allows easy characterization of toxic oligomeric aggregates [6,11,12].

Several studies have shown that sCT, in analogy with other amyloid proteins that show aggregative behavior, is toxic to cells in culture [12,13,14], although it does not appear to be directly involved in any neurodegenerative disease. However, unlike other amyloid proteins, sCT is characterized by a slow aggregation rate [4,13]. This property makes it particularly suitable to be used as a tool to study the molecular mechanisms of amyloid protein formation and neurotoxicity, with interest in the early stages of aggregation during which PFOs are formed as well as their interaction with cell membranes [6].

Many studies have indicated that the neuronal membrane composition and its chemical microenvironment play a fundamental role; for example, Lipid Rafts (LRs) have been shown to be involved in amyloidogenesis, in the protein aggregation process and in the mechanisms of interaction between cell membranes and amyloid proteins, thus contributing to their neurotoxic effect [15,16,17]. 

The monosialotetrahexosylganglioside (GM1) is one of the main components of LRs [18], and it has been indicated as the preferred target of PFOs [19,20]. In fact, its involvement in the interaction between Aβ oligomers and membranes, as well as between sCT oligomers and liposomes, is known [12,21,22]. In particular, studies carried out on artificial membranes have shown that the sCT administration in the environment promotes a calcium ionic current through these artificial membranes [11]. Furthermore, it has been observed that, as a result of the binding with the membranes, sCT undergoes a conformational change in *β*-sheet, and that depressions formed in the liposomal membrane could be calcium-permeable pores [11]. Based on this hypothesis, the number and conductance of these pores would cause intracellular calcium dysregulation, resulting in neuritic and synaptic changes [5]. 

In this regard, it has been shown that, in the presence of GM1, Aβ oligomers induce a reduction in Long-Term Potentiation (LTP), the electrophysiological phenomenon related to learning and memory processes, in hippocampal mouse slices [23,24,25]. A similar behavior has also been observed for sCT oligomers [12]. In particular, in our previous work we demonstrated that PFO-enriched sCT samples completely abrogated LTP in mouse hippocampal slices 80 min after the tetanic stimulation, unlike native monomer-enriched solutions that had no influence on synaptic plasticity, even compared to the control [12]. We hypothesized that the observed LTP impairment and neurotoxicity may depend on early membrane damage, induced by sCT PFOs but not by monomers, triggering an abnormal N-methyl-D-aspartate (NMDA) receptor-mediated Ca^2+^-influx [12]. It is known that the LTP induction requires synaptic activation of NMDA receptors, a subtype of glutamate receptor that is permeable to Na^+^, K^+^ and Ca^2+^; whereas during basal synaptic transmission (BST), glutamate released from the presynaptic axon terminal acts on alpha-amino-3-hydroxy-5-methyl-4-isoxazolepropionic acid (AMPA) receptors, which are permeable to Na^+^ and K^+^ and are primarily responsible for excitatory hippocampal neurotransmission [26].

Since our results were very similar to those reported for Aβ, we hypothesized that the LTP reduction induced by sCT oligomers was due to the interaction between PFOs and GM1 in LRs [12]. In fact, GM1 is known to be composed of four neutral sugar molecules and a negatively charged sialic acid residue [27]. It has been proposed that electrostatic interaction may be responsible for the initial contact between PFOs and GM1, followed by the membrane insertion to reduce hydrophobic mismatch [22].

Therefore, the use of substances capable of removing sialic acid from gangliosides, both in cells and in hippocampal slices, could be a strategy to assess the role played by the electrostatic interactions in the early stages of interaction of PFOs with LRs, thereby protecting against the deleterious effects of amyloid toxicity [5,28,29]. Among these substances, Neuraminidase (NAA) is a widely used enzyme due to its ability to cut sialic acid residues from membrane glycoconjugates. Notably, in 2001 Wang et al. produced a mixture of the enzyme from *Vibrio cholera* and *Arthrobacter ureafaciens* to reduce the sialic acid content of GM1 ganglioside in two cell lines, PC12 and SH-SY5Y [30]. The same procedure was also used in 2012 by Bucciantini and colleagues in H-END cells [31] and, more recently, by Oropesa-Nuñez et al. to study the effects of sialic acid cutting on the binding of toxic HypF-N oligomers to plasma membranes [32]. Therefore, based on these considerations, the aim of our work was to (i) verify whether the treatment of NAA could counteract the neurotoxic action induced by sCT oligomers in the HT22 cell line by increasing cell survival and (ii) assess whether NAA could have a partial or total protective action on synaptic transmission and plasticity.

## 2. Results

### 2.1. Effects of Treatment with NAA

**Figure 1:**
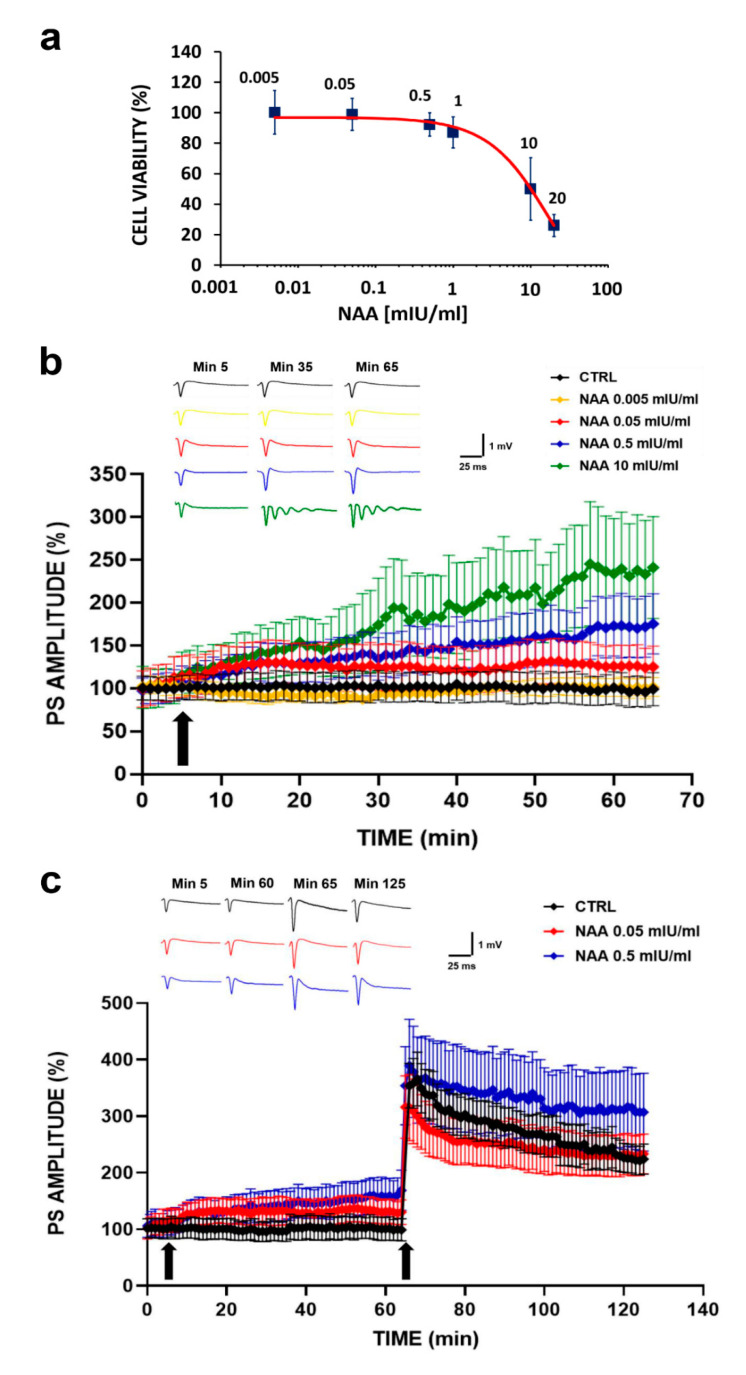
(**a**) Cell viability assessment in HT22 cells treated with increasing concentrations of Neuraminidase (NAA): the dose-response curve shows that in the concentration range between 0.005 and 0.5 mIU/mL, cell viability is not affected by treatment. A progressive reduction in cell viability is observed at higher concentrations, reaching the half maximal Inhibitory Concentration (IC50) at the dose of 10 mIU/mL. (**b**) Basal synaptic transmission (BST) in CA1 subfield of mouse hippocampal slices following NAA administration: % Population Spike (PS) amplitude as a function of time after NAA administration at different concentrations, applied at time 𝑡 = 5 min (arrow), is shown in mouse hippocampal slices of five experimental groups (black line, CTRL *n* = 6; yellow line NAA 0.005 mIU/mL, *n* = 5; red line NAA 0.05 mIU/mL, *n* = 6; blue line NAA 0.5 mIU/mL, *n* = 6; green line NAA 10 mIU/mL, *n* = 4). The insert shows representative recordings obtained from slices of each experimental group; curves of each group refer to PS at times 5, 35 and 65 min. (**c**) Synaptic plasticity in CA1 subfield of mouse hippocampal slices following NAA administration: %PS amplitude as a function of time after tetanic stimulation (HFS), applied at time 𝑡 = 65 min (arrow), following NAA administration at different concentrations (𝑡 = 5 min, arrow) is shown in mouse hippocampal slices of three experimental groups (black line CTRL, *n* = 6; red line NAA 0.05 mIU/mL, *n* = 7; blue line NAA 0.5 mIU/mL, *n* = 7). The insert shows representative recordings obtained from slices of each experimental group; curves of each group refer to PS at times 5, 60, 65 and 125 min.

To estimate the dose of NAA for which non-toxic effects are detected, we constructed a dose-response curve by treating HT22 cells with increasing concentrations of the substance and then assessing cell viability by means of 3-(4,5-dimethylthiazol-2-yl)-5-(3-carboxymethoxyphenyl)-2-(4-sulfophenyl)-2H-tetrazolium (MTS) assay (Figure 1a). As it can be observed, up to a dose of 0.5 mIU/mL, the treatment did not affect cell viability, whereas at higher concentrations, a progressive reduction was observed. It is worth noting that we obtained the half maximal Inhibitory Concentration (IC50) at a dose of 10 mIU/mL. 

Based on this evidence, we analyzed the effects of NAA on the synaptic transmission in the CA1 region of mouse hippocampal slices. The results, reported in Figure 1b, show how BST was differently modulated depending on the administered NAA concentrations, with a dose-response effect similar to that obtained with cell cultures. In particular, we observed that the treatment with the lowest concentration (0.005 mIU/mL) had no effect on BST, since the Population Spike (PS) amplitude values overlapped the control values. On the contrary, the treatment with the highest concentration (10 mIU/mL) induced a significant increase in BST. However, this concentration caused the onset of an epileptic trend a few minutes after the substance administration, confirming the results shown in the dose-response curve (IC50 = 10 mIU/mL). Regarding the intermediate NAA concentrations (0.05 and 0.5 mIU/mL), we observed that the treatment induced a BST modulation characterized by an increase in PS amplitude values, compared to the control, proportional to the concentration (PS amplitude values recorded for each group of mice slices at various times after NAA administration are reported in Table 1, where the values of statistical significance are also shown).

Based on the previous results, we assessed whether the NAA administration at intermediate concentrations (0.05 and 0.5 mIU/mL) could also modulate LTP, which is the electrophysiological paradigm of learning and memory processes. Results are summarized in Figure 1c. 

We note that, in addition to the increase in BST compared to the control already observed in Figure 1b, we did not find any effect on the synaptic plasticity expression induced by the NAA treatment. In fact, 65 min after the tetanic stimulation, the PS amplitude values of the group treated with 0.05 mIU/mL NAA were equal to those recorded for the control group (within the experimental errors), both in the LTP induction and maintenance phase. In the group treated with 0.5 mIU/mL NAA, we observed that the LTP induction phase was comparable on that of the control group, while the LTP maintenance phase was significantly enhanced (PS amplitude values recorded for each group of mice slices at various times after NAA administration are reported in Table 2, where the values of statistical significance are also shown).

In summary, the treatment with the two tested NAA concentrations did not induce any LTP reduction; in addition, the highest concentration seemed to further potentiate the long-term response.

### 2.2. Effects of the Combined Treatment with NAA and sCT PFOs

**Figure 2:**
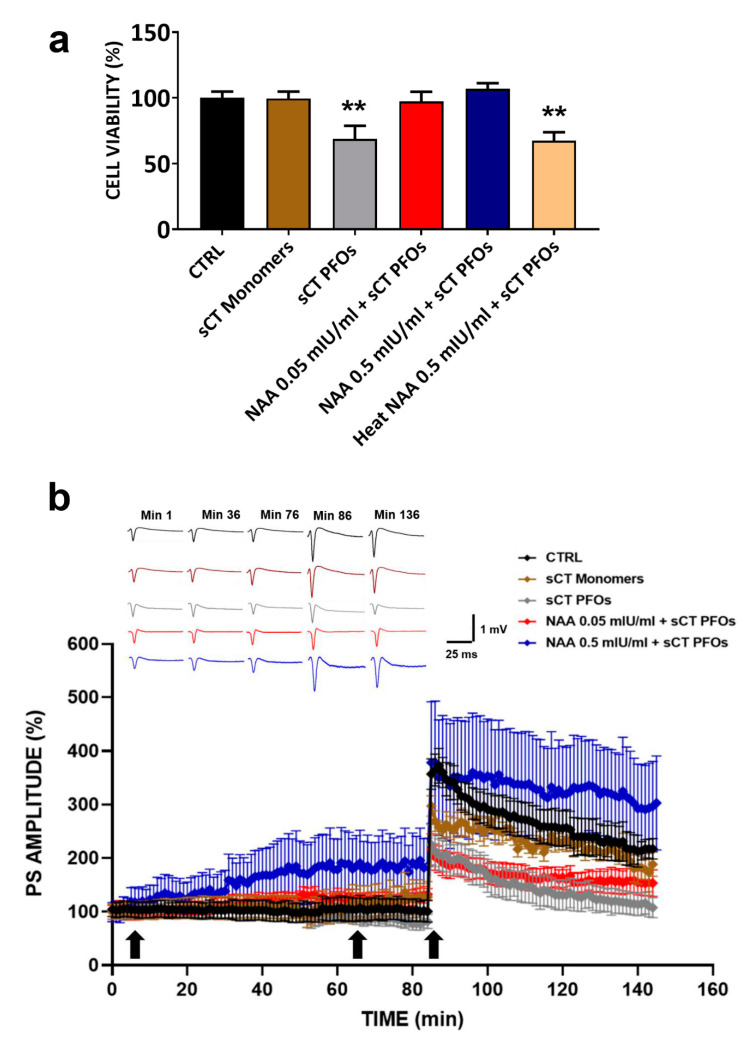
(**a**) Cell viability assessment in HT22 cells treated with Neuraminidase (NAA) and salmon Calcitonin (sCT): HT22 cells pre-treated with NAA at concentrations of 0.05 mIU/mL (red bar, *n* = 21 from N = 5 experiments) and 0.5 mIU/mL (blue bar, *n* = 16 from N = 4 experiments) for 1 h and then incubated with Prefibrillar Oligomers of salmon Calcitonin (sCT PFOs) for 24 h showed cell viability comparable to that of the CTRL group (black bar, *n* = 34 from N = 5 experiments). Treatment with sCT PFOs (grey bar, *n* = 20 from N = 5 experiments) significantly reduced cell survival by approximately 30% (** *p* < 0.01), whereas treatment with sCT monomers (brown bar, *n* = 17 from N = 4 experiments) did not affect cell viability. Finally, pre-treatment with heat NAA 0.5 mIU/mL + sCT PFOs (pink bar, *n* = 14 from N = 3 experiments) induced a reduction in cell viability of approximately 30%, like that obtained when HT22 cells were treated with sCT PFOs (** *p* < 0.01). (**b**) Synaptic plasticity in CA1 subfield of mouse hippocampal slices following NAA and sCT administration: % Population Spike (PS) amplitude as a function of time after tetanic stimulation (HFS), applied at time 𝑡 = 86 min (arrow), following NAA (𝑡 = 5 min, arrow) and sCT (𝑡 = 65 min, arrow) administration, is shown in CTRL (black bar, *n* = 6), in sCT monomers (brown bar, *n* = 4), in sCT PFOs (grey bar, *n* = 4), in NAA 0.05 mIU/mL + sCT PFOs (red bar, *n* = 7), and in NAA 0.5 mIU/mL + sCT PFOs (blue bar, *n* = 5) mice slices at minutes 1, 36, 76, 86 and 136. The insert shows representative recordings obtained from slices of each experimental group; curves of each group refer to PS at times 1, 36, 76, 86 and 136 min.

To determine whether removal of negatively charged sialic acid could counteract the toxic effects induced by treatment with sCT PFOs, HT22 cells were pre-treated with NAA (0.05 and 0.5 mIU/mL) for 1 h and then incubated with sCT PFOs for 24 h. We compared the obtained results with data from untreated HT22 cells (CTRL) and HT22 cells treated only with sCT PFOs or sCT monomers. As shown in Figure 2a, cells pre-treated with NAA at both concentrations show cell viability comparable to that of the control group. In contrast, treatment of the cells with sCT PFOs significantly reduced cell survival by approximately 30% (** *p* < 0.01). Further confirmation of this toxicity is provided by the result obtained following treatment of HT22 cells with thermally inactivated NAA. In fact, without the protective action of the enzyme, cell survival values were comparable to those obtained by treating the cells only with sCT PFOs (** *p* < 0.01). Interestingly, treatment with sCT monomers did not affect cell viability, further confirming the role of toxic species for oligomeric aggregates.

To assess the protective action of NAA on sCT PFO-induced amyloid neurotoxicity and on synaptic transmission, we pre-treated mouse hippocampal slices with NAA at the two concentrations previously tested and found to be not damaging (0.05 and 0.5 mIU/mL) for 60 min. Subsequently, we administered sCT PFOs or sCT monomers for 20 min. Finally, we compared the obtained results with those relative to untreated samples and to the two groups treated only with sCT. Results are shown in Figure 2b. 

Regarding BST, we observed that the trends of the PS amplitude values slightly increased after treatment with sCT monomers and slightly decreased after treatment with sCT PFOs. On the other hand, in mouse hippocampal slices pre-treated with NAA and subsequently treated with sCT PFOs, we observed a positive modulation of BST in both experimental groups, although with statistically significant PS amplitude values only for higher NAA concentration (0.5 mIU/mL). Regarding LTP, the PS amplitude values recorded for both sCT monomer and PFO treatment confirmed our previously published data, which showed that sCT PFOs abrogate LTP while sCT monomers are ineffective [12]. 

Furthermore, we can observe in Figure 2b that the pre-treatment of mouse hippocampal slices with NAA induced different effects on LTP, depending on the concentration. At the lowest concentration (0.05 mIU/mL), LTP was reduced in a similar way to that found after treatment with sCT PFOs alone. Conversely, at the highest concentration (0.5 mIU/mL), the LTP induction phase showed PS amplitude values similar to the control, while the maintenance phase settled at higher values (statistical significance) and remained constant over time (PS amplitude values recorded for each group of mice slices at various times after NAA and sCT administration are reported in Table 3, where the values of statistical significance are also shown.

## 3. Discussion

In our previous work we demonstrated, for the first time, that sCT PFOs exert a powerful neurotoxic effect on both cell viability and synaptic plasticity through an innovative mechanism where the two known paradigms, which are “membrane permeabilization” and “receptor-mediated”, must coexist [12]. Moreover, we recently proposed that the amyloid neurotoxicity process could be triggered by the electrostatic interaction between the positive PFOs and the negatively charged sialic acid of GM1 occurring in the outer part of the membranes [22].

Based on this evidence, the aim of our work was to investigate whether the neurotoxic action induced by sCT PFOs in HT22 cell lines and in mouse hippocampal slices could be counteracted by treatment with NAA, an enzyme able to remove the sialic acid residues from gangliosides.

### 3.1. Effects of Treatment with Different NAA Concentrations on Cell Viability and Synaptic Transmission

The definition of a dose-response curve was fundamental in determining the concentrations at which NAA does not itself exert a toxic effect on the experimental models used. In particular, we observed that the treatment of HT22 cells with NAA for 1 h did not impair cell viability in the concentration range of 0.005 to 0.5 mIU/mL (Figure 1a). On the contrary, the administration at higher concentrations resulted in a progressive reduction of cell survival, reaching IC50 at a dosage of 10 mIU/mL.

Based on the survival results, we evaluated the effect on BST in mouse hippocampal slices of the three non-toxic concentrations (0.005, 0.05 and 0.5 mIU/mL) and the concentration corresponding to the IC50 (10 mIU/mL) (Figure 1b). We observed that the gradual increase in concentration induced a proportional increase in the PS amplitude values. Specifically, at the lowest concentration (0.005 mIU/mL), values were similar to those of the untreated control group, while at the highest concentration (10 mIU/mL), there was an abnormal increase in BST related to the onset of an epileptic trend. These observations suggest that the lowest NAA dosage (0.005 mIU/mL) would probably not have been sufficient for an effective and complete cut of the sialic acids, while the high dosage causes neuronal damage.

Since the results obtained from the electrophysiological recordings confirmed the cell survival data, we decided to evaluate the effect of NAA administration on LTP at intermediate concentrations (0.05 and 0.5 mIU/mL) (Figure 1c). We observed that PS amplitude values in both NAA-treated groups were overlapping of those in the untreated control group. However, for mouse hippocampal slices treated with 0.5 mIU/mL, we detected a significant increase in PS amplitude values approximately 60 min after HFS. We note that LTP results are in line with those obtained from electrophysiological recordings of BST, confirming that NAA at intermediate concentrations (0.05 and 0.5 mIU/mL) does not exert a neurotoxic action but ensures full and complete preservation of the synaptic plasticity. 

In summary, we obtained optimal results at intermediate NAA concentrations (0.05 and 0.5 mIU/mL) in the treated groups compared to the untreated control, with a consistent increase in synaptic transmission both in terms of BST and LTP.

### 3.2. NAA Protection Effect from Amyloid Neurotoxicity Induced by sCT PFOs

The aim of our work was to test whether NAA treatment could counteract the neurotoxic action induced by sCT PFOs by removing negatively charged sialic acid from GM1. Therefore, HT22 cells and mouse hippocampal slices were subjected to a combined treatment with NAA at non-toxic concentrations (0.05 and 0.5 mIU/mL) and sCT PFOs. In addition, to confirm the role of amyloid oligomers as toxic species, we also evaluated the effect of sCT monomers in terms of cell survival and synaptic plasticity. Notably, we observed that pre-treatment of HT22 cells with NAA at both concentrations provides complete protection against sCT PFO-induced neurotoxicity, without any impairment of cell viability (Figure 2a). Furthermore, to validate the protective efficacy of NAA, we pretreated HT22 cells with the heat-inactivated enzyme and subsequently with sCT PFOs. Not surprisingly, cell viability was impaired similarly to what was observed with treatment with sCT PFOs alone. This result confirms the pivotal role played by GM1 in the mechanism of action of the amyloid PFOs on the cell membrane and, in particular, the electrostatic nature of the events triggering neurotoxicity. Thus, the cutting of sialic acids, obtained through NAA pre-treatment, seems to be an effective method to ensure complete protection from amyloid neurotoxicity. In the treatment of HT22 cells with sCT monomers, we found no impairment of cell viability, confirming the non-toxicity of sCT monomer species.

The results obtained from electrophysiological recordings on mouse hippocampal slices showed that pre-treatment with NAA followed by the administration of sCT PFOs induced different effects on synaptic transmission depending on the concentration used (Figure 2b). In particular, slices pre-treated with 0.05 mIU/mL responded to tetanic stimulation with a significantly depressed LTP compared to the untreated control group. This is especially true for the LTP induction phase, where PS amplitude values were comparable to those of slices treated only with sCT PFOs, suggesting that both experimental groups suffered significant damage. However, the slices subjected to the combined treatment with NAA 0.05 mIU/mL and sCT PFOs showed more stable and constant PS amplitude values over time in the LTP maintenance phase than the group treated with sCT PFOs only. This suggests that NAA, at the concentration of 0.05 mIU/mL, exerts a protective action against neurotoxic damage; however, this does not seem to be sufficient to completely preserve brain and cognitive function. On the contrary, slices pretreated with 0.5 mIU/mL showed an LTP induction phase comparable with that observed in the untreated control and a maintenance phase with higher PS amplitude values. 

In summary, results concerning electrophysiological recordings performed on mouse hippocampal slices suggest that the sialic acid removal obtained with NAA at a concentration of 0.05 mIU/mL is not sufficient for complete protection against sCT PFO-induced neurotoxicity, while a concentration ten times higher (0.5 mIU/mL), appears to provide more efficient sialic acid removal, reducing the attachment of sCT PFOs to the membrane surface. Regarding the treatment of mouse hippocampal slices with sCT monomers, we observed no impairment of the synaptic transmission. On the contrary, we detected an increase in BST comparable with that found in the group treated with 0.05 mIU/mL NAA and sCT PFOs. Following HFS, we obtained PS amplitude values comparable to those of the untreated control group, confirming the non-toxicity of the monomeric calcitonin species. 

We note that in a recent work we suggested that the interaction between PFOs and biological membranes depends on the electrostatic interaction among the negative charges of sialic acids and the positive charges of amyloid aggregates [22]. Moreover, it is known that hippocampal synaptic transmission is mediated by AMPA glutamate ionotropic receptors, which are particularly sensitive to NAA treatment [28]. In this regard, it has been reported that NAA treatment of rat neuronal membranes increase the binding affinity of glutamate to its receptor. Specifically, it has been proposed that the removal of negatively charged sialic acids from the surface of neuronal membranes can minimize electrostatic repulsion between the glutamate and membranes, leading to an increase in concentration near the receptor [28]. 

Based on the above considerations and the experimental results shown in this work, we propose that NAA treatment leads to an alteration of the surface charges of LRs, preventing the interaction between sCT PFOs and GM1. 

This hypothesis is supported by the PS amplitude values obtained from electrophysiological recordings of the BST, which increase proportionally to the NAA dosage used, probably due to an improved interaction between glutamate and AMPA receptors as previously proposed [28]. The same phenomenon could also explain the increase in PS amplitude values in the LTP maintenance phase that we observed only in hippocampal slices treated with 0.5 mIU/mL NAA. In fact, it is known that, following HFS, there is a redistribution in the membrane of AMPA receptors, which are normally stored in synaptic vesicles [33]. Thus, we speculate that efficient sialic acid cutting, achieved by NAA treatment at 0.5 mIU/mL, promotes enhanced interaction between glutamate and AMPA receptors.

**Figure 3:**
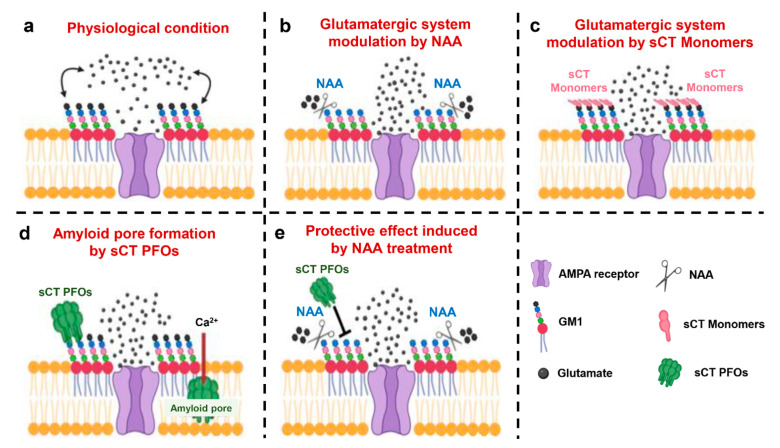
A novel model to explain the role of electrostatic interactions involved in the mechanism of action of calcitonin oligomers and monomers. (**a**) Physiological condition: the negative charges of the sialic acid contained in the monosialotetrahexosylganglioside (GM1) generate an electrostatic field that partially repels the anionic neurotransmitter glutamate, promoting fine tuning of the glutamatergic system. (**b**) Modulation of the glutamatergic system by Neuraminidase (NAA): the action of NAA removes negatively charged sialic acid residues from GM1, resulting in an alteration of the membrane surface charges and in the increase of the relative concentration of the anionic neurotransmitter glutamate near to the alpha-amino-3-hydroxy-5-methyl-4-isoxazolepropionic acid (AMPA) receptor. (**c**) Modulation of the glutamatergic system by salmon Calcitonin (sCT) monomers: the binding of positive sCT monomers with GM1 masks the negative charges of the sialic acid residues, reducing the electrostatic repulsion between the anionic neurotransmitter glutamate and the membrane. (**d**) Formation of the amyloid pore by Prefibrillar Oligomers of salmon Calcitonin (sCT PFOs): the electrostatic interaction between the positive charges of sCT PFOs and the negative charges of GM1 drives the oligomer insertion into the membrane, resulting in the amyloid pore formation and toxicity. (**e**) Protective effect induced by NAA treatment: removal of negatively charged sialic acid residues from GM1 by NAA prevents electrostatic interaction between sCT PFOs and membrane counteracting amyloid pore formation and neurotoxicity.

In agreement with this hypothesis, we also observed an increase in BST following sCT monomer administration in mouse hippocampal slices. Indeed, the interaction between the positive charges of sCT monomers and the negative charges of sialic acids could reduce the electrostatic repulsion between anionic neurotransmitter glutamate and neuronal membranes near AMPA receptors, thus promoting an increase in receptor-ligand interactions (our hypothesis is summarized in Figure 3).

## 4. Materials and Methods

### 4.1. Animals

Thirty male mice aged 6 to 9 weeks old, belonging to the strain BALB/c mice, were used according to the procedures established by the European Union Council Directive 2010/63/EU for animal experiments [34]. All the experimental protocols were performed after approval of the project by the Italian Ministry of Public Health (authorization No. 86/2018-PR).

### 4.2. Cell Cultures

HT22 cells were developed from their analogous HT4 cells, immortalized from primary mouse hippocampal neurons. HT22 cells were maintained at 37 °C, 10%, CO_2_ in Dulbecco’s Modified Eagle’s Medium (DMEM, Sigma Aldrich–D6546) supplemented with 10% heat-inactivated fetal bovine serum (FBS) and kept at less than 50% of confluence. Differentiation was carried out in Neurobasal Medium (NBM, Gibco, 21103-49) containing N2 supplement (Gibco-17502048) for at least for 24–48 h before use.

### 4.3. sCT Sample Preparation by Size Exclusion Chromatography (SEC)

Solutions enriched with 1mM sCT monomers were prepared by dissolving sCT lyophilized powder (European Pharmacopoeia, EDQM, France) in desalted water. To limit the aggregation process, the solution was quickly frozen and stored at −80 °C. Aggregated sCT native solutions were prepared by incubating 2 mg of sCT powder in 5 mM phosphate buffer (PB: PB 5 mM, pH 7.4) at room temperature overnight. The solution was then loaded into the SEC column to purify oligomeric species enriched fractions [4]. In brief, samples were loaded in Sephadex G50-SEC column (GE HEALTHCARE, Milano, Italy—height: 500 mm, section: 20 mm). The column, maintained at 4 °C, was pre-equilibrated at the same ionic strength as the samples and calibrated with a solution containing standards: BSA 1 mg (66 kDa), Cytochrome c 1 mg (12.4 kDa-Combithek Boehringer, Mannheim, Germany), Aprotinin 1 mg (6.5 KDa) and Somatostatin 1 mg (1.63 KDa), suspended in 5 mM PB buffer pH 7.4 and centrifuged at 15.700 *g* × 10 min. Monomeric or aggregated sCT native solutions (0.5 mL aliquots) were eluted in the column monitoring absorption at 280 nm by a variable wavelength UV detector (BIORAD Econo UV monitor, Hercules, CA, USA). Fractions collected (Gilson FC 203B, 1.4 mL/fraction) were administered directly in cell cultures to test their effects on cellular viability and in mouse hippocampal slices to evaluate synaptic plasticity modulation. 

### 4.4. NAA Solution Preparation

Cell surface sialic acid depletion was achieved by treatment with NAA. HT22 cells were incubated in Hanks’ Balanced Salt Solution (HBSS) (Life technologies) with Vibrio cholera (78%) and *Arthrobacter ureafaciens* (22%) NAA for 1 h at 37 °C [30]. The stock solution (200×, containing 156 mIU/mL of NAA *Vibrio cholerae* and 44 mIU/mL of NAA *Arthrobacter ureafaciens*, was adequately diluted to reach the desired concentrations. Control cells were treated identically, except for the presence of peptide. In addition, a further control group was obtained by deactivating the enzyme by heat treatment at 70 °C for 15 min.

### 4.5. Assessment of Cell Viability by MTS Assay

Cell viability was assessed using CellTiter 96 AQ_ueous_ One (Promega, USA), which is a colorimetric method to determine the number of viable cells in proliferation or chemosensitivity assay. The CellTiter 96 AQ_ueous_ Assay is composed of a novel tetrazolium compound, (3-(4,5-dimethylthiazol-2-yl)-5-(3-carboxymethoxyphenyl)-2-(4-sulfophenyl)-2H-tetrazolium—MTS) and an electron-coupling reagent (phenazinemethosulfate—PMS). MTS is bioreduced by cells into a formazan product that is soluble in tissue culture medium. The absorbance of the formazan at 490 nm can be measured directly from 96-well assay plates without additional processing. In brief, 20 µL of MTS/PMS solution was added to 100 µL of HBSS in each well and incubated for at least 2 h at 37 °C. The recommended concentrations of MTS solution and PMS solution were optimized for a wide variety of cell lines grown in 96-well plates containing 100 µL of medium per well. This resulted in final concentrations in the assay of 333 µg/mL MTS and 25 µM PMS. The conversion of MTS aqueous, soluble formazan is accomplished by dehydrogenase enzymes found in metabolically active cells. The quantity of formazan product as measured by the amount of 490 nm (Spark Multimode Microplate Reader, Tecan, Austria) absorbance is directly proportional to the number of living cells in culture.

### 4.6. Extracellular Recordings in Mouse Hippocampal Slices

BST and LTP were examined in the Schaffer collateral/commissural CA1 pathways in mouse hippocampal slices prepared according to conventional procedures [35]. All efforts were made to minimize the number of animals used and their suffering. Under anesthesia with halothane (2-Brom-2-chlor-1,1,1-trifuor-ethan), they were sacrificed and their brains were quickly removed and placed in cold, oxygenated artificial cerebral spinal fluid (ACSF) containing the following (in mM): NaCl 124, KCl 2, KH_2_PO_4_ 1.25, MgSO_4_ 2, CaCl_2_ 2, NaHCO_3_ 26, and glucose 10. The hippocampus was rapidly dissected, and slices (450 μm thick) were cut transversely with a McIlwain tissue chopper (Mickle Laboratory Engineering Co., Gomshall, UK) and transferred into a tissue chamber, where they were laid in an interface between oxygenated ACSF and humidified gas (95% O_2_, 5% CO_2_) at 32–34 °C (pH = 7.4), constantly superfused at flow rate of 1.2 mL/min. Extracellular recordings of the population spikes (PSs) were made in the stratum pyramidale of the CA1 subfield with glass microelectrodes filled with 2 M NaCl (resistance 5–10 MΩ). Orthodromic stimuli (10–500 mA, 20–90 ms, 0.1 Hz) were delivered through a platinum electrode placed in the stratum radiatum in the Schaffer collateral/commissural CA1 pathways. The test stimulus intensity of 50 ms square pulses was adjusted to elicit a PS of 2–3 mV at 0.03 Hz. PS amplitude was calculated every minute as the average of six recordings performed every 10 s.

To exploit BST, the PS was recorded for 1 h. After recording stable signals (20–30 min), the hippocampal slices were treated with NAA solution and/or monomer- or PFO-enriched solutions of sCT to assay their effects on synaptic transmission. PFOs and monomers were diluted in carboxygenate ACSF at a final concentration of about 2 μM, which was subsequently used to perfuse the slices. Then, a tetanic stimulation (100 Hz, 1 s) was delivered to induce LTP at the same stimulus intensity used for the baseline responses. Field potentials were fed to a computer interface (Digidata 1440A, Axon Instruments, Foster City, CA, USA) for subsequent analysis with the software PCLAMP10 (Axon Instruments).

### 4.7. Statistical Analysis

Statistical analysis was performed using GraphPad Prism 8 Software (Prism 8.0.1, La Jolla, CA, USA). Cellular viability estimations for each experimental condition were obtained in quadruplicate, and data were normalized with respect to controls. A multiple comparison in cellular viability was obtained by ANOVA and the Dunnett Test, with a confidence level of 95% and 99%. For electrophysiological experiments, data were expressed as mean ± SEM, and n represented the number of slices analyzed. Data were compared with ANOVA and Tukey’s Multiple Comparison Test and were considered significantly different if *p* < 0.05.

## 5. Conclusions

Based on the scenario described above, we proposed an innovative model where an electrostatic interaction between the positive charges of sCT PFOs and the negative sialic acid residues of gangliosides is responsible for the first contact between oligomers and membranes (Figure 3d). Subsequently, to minimize the hydrophobic mismatch, PFOs are inserted into the membrane forming amyloid pores leading to neurotoxicity. Treatment with NAA removes the negative charge of the sialic acid residues, reducing or preventing the interaction between sCT PFOs and the membrane and thus exerting a protective action against pore formation and neurotoxicity (Figure 3e). The alteration of membrane surface charges caused by NAA treatment (Figure 3b), as well as the masking induced by sCT monomers (Figure 3c), results in a positive modulation of the hippocampal glutamatergic system by promoting receptor-ligand interaction. This effect seems to be proportional to the NAA concentration, and an optimum value seems to be 0.5 mIU/mL.

We believe that our results could be important and innovative for the investigation of the molecular mechanisms of toxicity exerted by amyloid proteins and for the development of new therapeutic targets aimed at counteracting the detrimental action exerted by amyloid oligomers.

## Figures and Tables

**Table 1:** Percentage of PS amplitude values of BST recorded in the CA1 region of hippocampal slices from the control group and groups treated with different concentrations of NAA at different times.

TIME (min)	CTRL (PS% Amplitude)	NAA 0.005 mIU/mL (PS% Amplitude)	NAA 0.05 mIU/mL (PS% Amplitude)	NAA 0.5 mIU/mL (PS% Amplitude)	NAA 10 mIU/mL (PS% Amplitude)	SIGNIFICANCE between Groups at Different Times
**5**	99.4 ± 14.1	103.3 ± 11.0	112.8 ± 24.0	103.6 ± 18.4	109.8 ± 27.5	no significance
**35**	101.5 ± 16.9	94.0 ± 3.4	125.7 ± 28.1	146.1 ± 27.2	179.4 ± 52.1	CTRL vs. NAA 10 mIU/mL, *** *p* < 0.001
**65**	95.6 ± 17.5	100.5 ± 12.4	124.2 ± 22.8	171.9 ± 35.9	233.5 ± 62.6	CTRL vs. NAA 0.5 mIU/mL, *** *p* < 0.001; CTRL vs. NAA 10 mIU/mL, **** *p* < 0.0001

PS: Population Spikes; BST: Basal Synaptic Transmission; NAA: Neuraminidase; CTRL: control group; ***: *p* < 0.001; ****: *p* < 0.0001.

**Table 2:** Percentage of PS amplitude values of BST and LTP recorded in the CA1 region of hippocampal slices from the control group and groups treated with two different concentrations of NAA at different times.

TIME (min)	CTRL (PS% Amplitude)	NAA 0.05 mIU/mL (PS% Amplitude)	NAA 0.5 mIU/mL (PS% Amplitude)	SIGNIFICANCE between Groups at Different Times
**5**	100.5 ± 17.4	114.7 ± 20.9	106.9 ± 21.0	no significance
**60**	100.8 ± 20.9	130.7 ± 21.2	159.0 ± 33.5	CTRL vs. NAA 0.5 mIU/mL, ** *p* < 0.01
**66**	354.0 ± 39.3	316.3 ± 55.4	354.1 ± 69.1	no significance
**125**	224.3 ± 26.5	231.2 ± 36.3	307.4 ± 68.5	CTRL vs. NAA 0.5 mIU/mL, ** *p* < 0.01

PS: Population Spikes; BST: Basal Synaptic Transmission; LTP: Long-Term Potentiation; NAA: Neuraminidase; CTRL: control group; **: p < 0.01.

**Table 3:** Percentage of PS amplitude values of BST and LTP recorded in the CA1 region of hippocampal slices from the control group and groups treated with NAA and sCT at different times.

TIME (min)	CTRL (PS% Amplitude)	sCT Monomers (PS% Amplitude)	sCT PFOs (PS% Amplitude)	NAA 0.05 mIU/mL + sCT PFOs (PS% Amplitude)	NAA 0.5 mIU/mL + sCT PFOs (PS% Amplitude)	SIGNIFICANCE between Groups at Different Times
**1**	104.7 ± 13.9	100.3 ± 14.4	99.9 ± 9.7	99.6 ± 12.9	99.3 ± 18.0	no significance
**36**	102.0 ± 18.7	108.6 ± 21.7	105.7 ± 15.8	117.8 ± 12.9	156.2 ± 44.6	no significance
**76**	104.2 ± 18.6	128.7 ± 25.8	87.2 ± 16.9	124.4 ± 11.3	184.5 ± 52.8	CTRL vs. NAA 0.5 mIU/mL + sCT PFOs, *** *p* < 0.001
**86**	356.8 ± 28.6	297.3 ± 19.1	207.5 ± 38.6	216.1 ± 29.1	378.3 ± 113.8	CTRL vs. sCT PFOs and NAA 0.05 mIU/mL + sCT PFOs, **** *p* < 0.0001
**136**	226.4 ± 32.7	201.0 ± 12.7	105.5 ± 20.1	157.6 ± 22.9	313.9 ± 79.8	CTRL vs. sCT PFOs, **** *p* < 0.0001; CTRL vs. NAA 0.05 mIU/mL + sCT PFOs, ** *p* < 0.01; CTRL vs. NAA 0.5 mIU/mL + sCT PFOs, *** *p* < 0.001

PS: Population Spikes; BST: Basal Synaptic Transmission; LTP: Long-Term Potentiation; NAA: Neuraminidase; sCT: salmon Calcitonin; CTRL: control group; sCT PFOs: Prefibrillar Oligomers of salmon Calcitonin; **: *p* < 0.01; ***: *p* < 0.001; ****: *p* < 0.0001.

## Data Availability

The data presented in this study are available on request from the corresponding author.
